# ﻿A digitization workflow of dry-pinned collections of Lepidoptera

**DOI:** 10.3897/zookeys.1264.134756

**Published:** 2025-12-15

**Authors:** Laurel Kaminsky, Stacey Huber, Trudi Deuel, Ngoc-Nhu V. Tran, Erin Jane Howard, Aaron Leopold, Anupama Priyadarshini, David Plotkin, Akito Y. Kawahara

**Affiliations:** 1 Marston Science Library, Smathers Libraries, University of Florida, 444 Newell Drive, Gainesville, Florida 32608, USA University of Florida Gainesville United States of America; 2 McGuire Center for Lepidoptera and Biodiversity, Florida Museum of Natural History, University of Florida, 3215 Hull Road, Gainesville, Florida 32611, USA University of Florida Gainesville United States of America

**Keywords:** Butterfly, copy stand, digitization, imaging, moth, museum

## Abstract

Butterflies and moths (Lepidoptera) are one of the most commonly found insect groups in museum collections. Yet many specimens still lack digital data and few digitization workflows are available. Here we present a digitization workflow for natural history collections that can be widely applicable for any museum or dried pinned-specimen collection of Lepidoptera. Our workflow consists of pre-imaging preparation, usage of a copy stand for imaging pinned specimens and data labels, image processing, and transcription, the latter two which utilize Python scripts for optimization.

## ﻿Introduction

Arthropods are a diverse group of organisms with morphologically distinctive life stages (Giribet and Edgecomb 2012). There are an estimated 300 million adult arthropod specimens across 223 North American collections (Cobb et al. 2019). These specimens and their data are needed to answer questions in many fields of biology, ranging from the genetic level to the study of entire ecosystems (Short et al. 2018). Currently, the majority of data is inaccessible to researchers around the world because many collections are not digitized. Digitization creates a record of collections that enables global and widespread access to data, broadens the scale and scope of collections-based research, and enhances accessibility to natural history collections (NHCs) data (Nelson and Ellis 2018; Hedrick et al. 2020).

Methods that NHCs use for record keeping have changed over the years. Recording label data has historically focused on tracking specimens in a collection. Modern digitization approaches are focused on capturing and sharing label data, metadata, and research grade high resolution images of specimens in NHCs with researchers around the world through data aggregators such as Integrated Digitized Biocollections (iDigBio 2024) and Global Biodiversity Information Facility (GBIF 2024).

Due to these advancements, exponentially more data are being gathered from specimens to answer a diverse array of questions (Ball-Damerow et al. 2019). Specimen image and label data can be used to assemble species lists for specific regions (Terry et al. 2023), provide insight into biological phenomena such as biogeography (Bedford et al. 2012; Earl et al. 2021), phenology (Wells and Tonkyn 2014; Echevarría Ramos and Hulshof 2019; Li et al. 2019; Belitz et al. 2023), fungal pathogens (May and Ristaino 2004), climate change (Kharouba et al. 2019), morphology (Holmes et al. 2016; Owens et al. 2020; Hamilton et al. 2022), and behavior (Aiello et al. 2021; Childers et al. 2023). Images can be used to train neural networks to identify arthropods, as has been demonstrated in analyses of Hymenoptera (Buschbacher et al. 2020), Hemiptera (Popkov et al. 2022), and Lepidoptera (Almryad and Kutucu 2020; Cunha et al. 2023; Qin et al. 2024).

**Figure 1.**
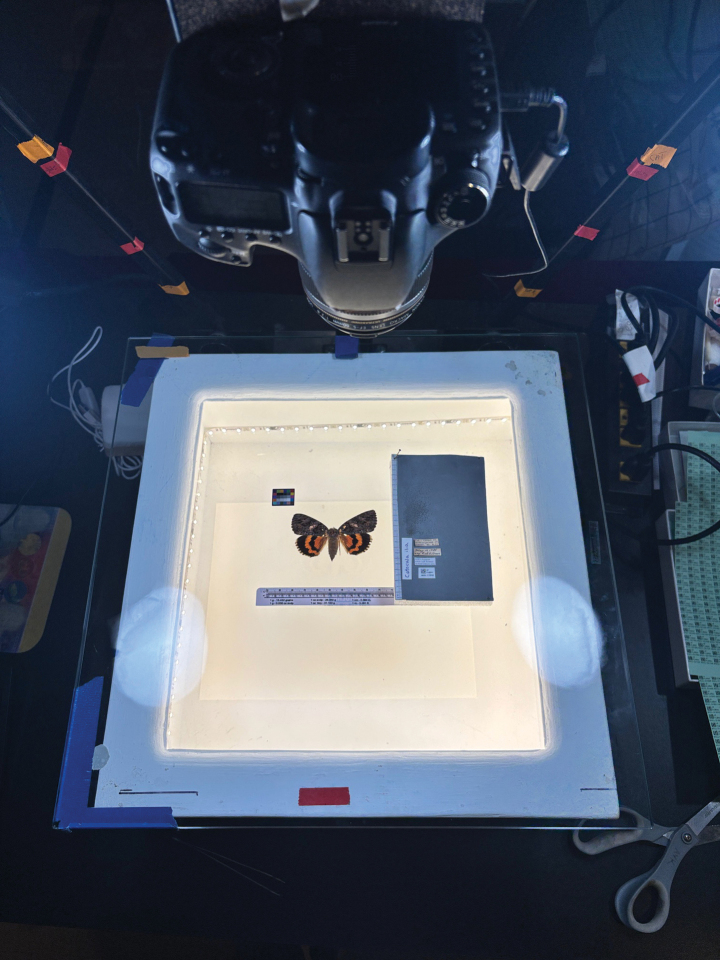
View of copy stand digitization from the perspective of the digitizer. The specimen was mounted in putty and placed on the tempered glass along with the color card. Label data is placed on the gray foam platform on the right along with the scientific name. The lightbox has internal LED lighting to minimize shadows. The copy stand lights are not shown but are located on both sides of the lightbox.

Digitization workflows usually consist of five steps: pre-imaging preparation, imaging, image processing, transcription, and georeferencing (Nelson et al. 2012). The steps of the digitization workflow depend on the organism method of preservation, the research question, and the NHC’s institutional protocols. Key features of most workflows are the use of a light source and a camera to image the specimen and/or data labels (Fig. 1). The copy stand is a widely used tool consisting of a flat platform with a central mount for adjusting camera height and integrated or separate lighting. It is particularly suited for photographing specimens preserved as flat sheets or packets, such as herbarium and fungarium specimens, as well as pinned arthropods (Nelson et al. 2015; Thiers et al. 2016; Harris and Marsico 2017; Takano et al. 2019).

In NHCs, the majority of arthropod dry collections remain undigitized (Cobb et al. 2019). Digitizing these collections and providing access to these data is challenging because of the sheer volume of specimens, diversity of life stages, and methods (e.g. pinned, papered, fluid) of preservation and storage. In addition, a pinned-arthropod specimen often obscures label data located beneath it on the pin. Arthropod digitization can be perceived as still being in its infancy but rapidly evolving. Digital records of specimens in arthropod collections typically consist of images of the specimens and transcriptions of associated label data.

For arthropods, there are few published peer-reviewed digitization workflows that use copy stands or scanners. One Lepidoptera workflow is the Natural History Museum’s iCollections project (Paterson et al. 2016; Blagoderov et al. 2017), which covers specimen selection, imaging, transcription, georeferencing, and data management. A copy stand variant workflow was used to digitize papered swallowtail butterflies (Caspers et al. 2019). Two fluid digitization papers on arthropods have been published. One was designed for stonefly digitization and entails imaging a specimen in a petri dish and associated data labels underneath (DeWalt et al. 2018). The second workflow utilized a flatbed scanner and 3D-printed boxes to image specimens and data labels for caddisflies in fluid (Mendez et al. 2018).

The vast number of specimens in many NHCs makes complete digitization challenging, especially for large and rapidly growing collections, when using commonly employed tools like a copy stand (Cobb et al. 2019). Some museums are now employing fast, automated methods that include conveyor belts (Tegelberg et al. 2014, 2017) which allow simultaneous preparation and imaging of specimens. Further automation of the copy stand digitization process, including imaging, transcription, and georeferencing, has been proposed for dry collections through the National Science Foundation (NSF)-funded LightningBug project (Hereld et al. 2017; Hereld and Ferrier 2019) and for capturing multispectral images of specimens (Chan et al. 2022). Similar advancements are being developed for fluid collections, enabling imaging without removing specimens from vials (Dupont et al. 2020).

Another approach to increase digitization speed is to image a whole insect drawer at once and parse single specimen images into separate records. Whole-drawer imaging usually captures an image of the specimen and associated label data. Several programs and workflows have been proposed, including InSelect (Hudson et al. 2015), GigaPan (Bertone et al. 2012), SATSCAN (Blagoderov et al. 2012; Mantle et al. 2012), and DSCAN (Schmidt et al. 2012). Some whole-drawer imaging workflows involve taking multiple images of a drawer, using software to stitch them together, and then separating the stitched image into a unique image and identifier for each specimen (Blagoderov et al. 2012; Mantle et al. 2012; Hudson et al. 2015). Other methods create a panorama of the whole drawer from individual images (Bertone et al. 2012). However, the problem with whole drawer imaging is that it does not usually capture associated label data because the data is either blocked by the specimen above or because labels are stacked on top of each other.

After imaging and image processing, data labels need to be transcribed. Transcription is the rate limiting step in the digitization process (Guralnick et al. 2024) because of limited time and money for personnel, tediousness of parsing information into different fields, difficult to read labels, and time to double check and catch transcription errors. The two most common transcription methods are to either type directly into a database or to use computer programs to help automate transcription. One widely used platform for transcription is Notes from Nature (NfN), which enables volunteers from around the world to transcribe records and gather scientific data on the Zooniverse platform (Hill et al. 2012). Each record is transcribed multiple times and the results of the transcribers can be cleaned, reconciled, and uploaded into a database.

Despite widespread use of copy stand and transcription workflows, published or publicly accessible workflows represent only a small portion of those used by entomological collections across the world. An increasing number of NHCs in the entomological community are sharing workflows on BugFlow, a community-driven resource available on Slack and GitHub (Entomology Collection Network 2023). Publishing workflows is crucial for disseminating best practices and promoting optimization, standardization, and reproducibility within and across institutions.

The Lepidoptera of North America Network (LepNet) project, funded by the NSF, enabled 29 research collections to digitize 2.1 million specimens covering most Lepidoptera species in North America, and it created large datasets of select species to study them in depth (Seltmann et al. 2017). Here we present the workflow for pre-imaging preparation, imaging, image post-processing, and transcription used for LepNet, implemented at the McGuire Center for Lepidoptera and Biodiversity (**MGCL**), which has one of the largest collections of Lepidoptera (Kawahara et al. 2012), The workflow used a copy stand to capture dorsal and ventral images and label data of pinned-Lepidoptera specimens, and a post-processing computer scripting pipeline for renaming and uploading images and label data onto the Symbiota Collections of Arthropods Network (**SCAN**).

## ﻿Materials and methods

There are four aspects that underpin our digitization protocol. 1) Image capture set up using a copy stand, lightbox, and camera; 2) Barcode label selection and application; 3) Running software and image processing scripts that streamline editing to ensure images have consistent quality and appearance; 4) Transcription of processed images. We experimented with using a copy stand to image fluid specimens and have included observations in Suppl. material 1.

### ﻿Image capture setup

The image capture setup requires five key pieces of equipment: a copy stand, lightbox, foam sheet, tempered glass, and a camera. A detailed list of supplies is provided in Suppl. material 2. We used a Beseler CS-14 copy stand with a custom-built lightbox, based on the design by Ortiz-Acevedo et al. (2020), placed on the copy stand as an imaging platform (Fig. 1). The lightbox features two strips of LED lighting along the inner walls to provide backlighting and minimize shadows. Its inner base was lined with a removable neutral gray surface, made from either craft foam or a metal plate. For enhanced contrast, white or black foam backgrounds were used for specimens with gray wing margins.

Photographs were taken with a Canon EOS7D camera, with a 60-mm lens for most specimens. Larger specimens, such as *Antheraea polyphemus* (Cramer), were imaged using a 18–55 mm lens. Image quality was optimized using camera features such as white balance, aperture, shutter speed, and ISO sensitivity. Midway through the project, we incorporated the Datacolor Spyder X Elite monitor calibration software (Datacolor, Lawrenceville, New Jersey, purchased 2021) to further standardize image lighting.

**Figure 2.**
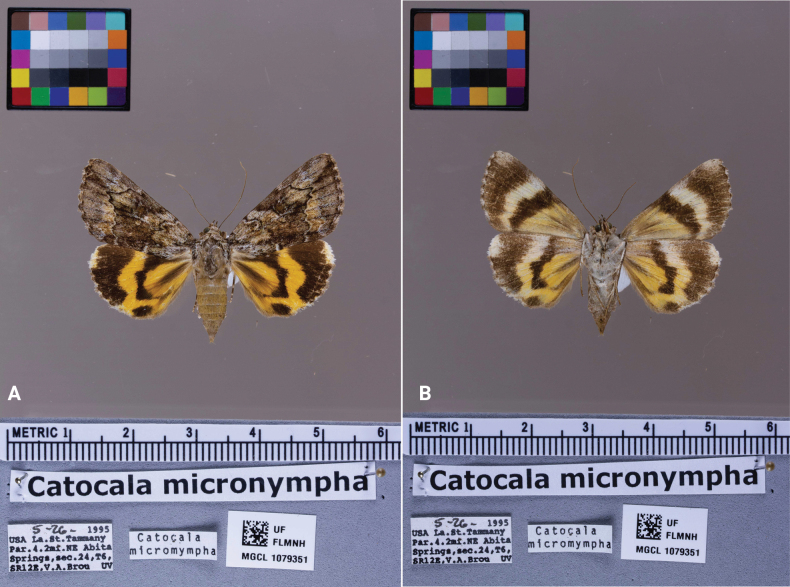
Example of the dorsal and ventral images of a digitized specimen (*Catocala micronympha* Guenée). Items in the picture include a color card (top left), ruler, scientific name, label data, and a unique barcode. A. Dorsal image; B. Ventral image.

Our protocol includes a color card, silicone putty, and gray foam board placed on top of tempered glass. The gray foam board was constructed by gluing a layer of gray foam over plank Styrofoam. Each component of the setup was placed in a standardized location. The color card was positioned on the glass so that it would appear in the top left corner of the image. The silicone putty used to hold the specimen was placed adjacent to the foam board, ensuring the foam board did not interfere with the specimen (Fig. 2). The data labels, barcode, ruler, and scientific name were arranged on the foam board.

### ﻿Barcode label selection and application

For pinned-specimen digitization, we either used a green colored paper barcode label, measuring 19 mm x 13 mm, printed at MGCL with BarTender 10.1 (Seagull Scientific, Bellevue, WA, USA) with code 128 or a 14 mm x 10 mm plastic laminated data matrix barcode from AlphaSystems (a Hunkar Technology Company, Midlothian, VA, USA). Each label included a human- and machine-readable unique identifier, to provide essential specimen tracking and standardize image file names.

### ﻿Running software and image processing scripts

The program EOS Utility version 2.11 (Canon, USA) was used to take .cr2 and .jpg images. Settings on the camera were F-stop: F/16, sensitivity to light (ISO-200), and exposure time (shutter speed 1/15 sec). For image processing, multiple custom scripts (Leopold and O’Connell 2023) were written in Python3 (Van Rossum and Drake 2009). These scripts can be used by other collections for the same tasks and/or have data matrix or code 128 barcodes. Scripts were for renaming images (“Datamatrix.py” script), reducing size of images (“Rescale.py” script), generating .csv file of images in current working directory to make a skeletal data file (“wls.py” script), and zipping images into folders for long term storage (“Zipper.py” script). The “Datamatrix.py” script (Leopold 2021) used the libraries “dmtx” (Misbakh-Soloviov 2016) and “zbar” (Brown 2012) to extract the barcode and rename the file. The “Rescale.py” script used the PIL module, specifically the submodules “Image” and “ExifTags”, to reduce the size and rotate the images (Clark 2021). The correct image orientation was determined by the value extracted from ExifTags, which was an integer between one and eight with one indicating zero degrees and eight indicating 90 degrees. The “wls.py” script was a skeletal file upload, which took the images in a file and generated a .csv file listing all file names. The scientific name was entered manually into the .csv file and uploaded onto SCAN. The “Zipper.py” script zipped specified images into a new folder of a specified size for long term archiving, sharing images with collaborators, or uploading onto SCAN.

### ﻿Transcription

Records were transcribed on NfN, a digital platform aimed at engaging citizen scientists from around the world (Hill et al. 2012). Each project is called an “Expedition.” Every specimen in the expedition must be transcribed by three people to be considered complete and the transcriptions were compiled into one .csv file. Transcriptions from NfN were cleaned then uploaded onto SCAN. Select records were also transcribed directly into SCAN.

## ﻿Results

### ﻿MGCL Digitization workflow

Our workflow consisted of four modules: 1) Pre-Imaging preparation; 2) Specimen imaging; 3) Image processing; 4) Transcription. Detailed documentation for pinned-specimen digitization is provided in Suppl. material 3. Pinned-specimen digitization workflow required 1–2 people to complete the following steps: 1. Removing the specimen from the drawer and preparing the specimen and label data for imaging. 2. Imaging. 3. Repinning data labels and data matrix barcode. 4. Returning the specimen to the drawer.

### ﻿Pre-imaging preparation of pinned specimens

A project plan and specimen selection were crucial to the success of digitization. A project plan communicates to collection users all details of what specimens will be digitized, project length, and outcomes, and where specimens are temporarily stored. The MGCL is a large facility, making coordination essential to avoid disruptions to other users. Specimen selection was guided by factors such as curation quality, researcher’s data needs, and institutional objectives. We moved drawers to a part of the collection near the digitization station to minimize traffic and use of the compactors. Labels with the scientific names of taxa, which were placed in the image of the corresponding species, were printed in advance.

### ﻿Pinned-specimen imaging

The imaging interface, EOS Utility, was opened and a destination folder for images was created. The imaging surface of the copy stand was cleaned. The camera was adjusted to the optimal height and the camera settings, including the shutter speed, ISO, and aperture were checked.

**Figure 3.**
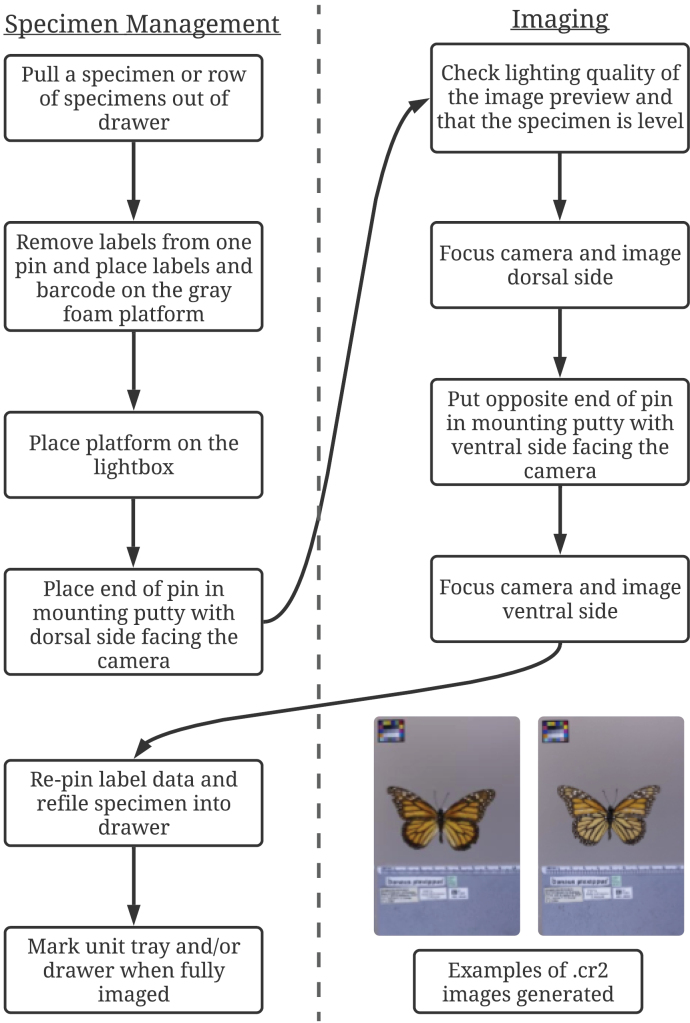
Digitization workflow for pinned specimens. Workflow consists of specimen management and imaging tasks. Arrows trace transitions between tasks. Digitization begins with removing the specimen from the box, pulling label data from the pin, placing it onto the gray foam block, and mounting pinned specimen in the putty. The dorsal and ventral images are taken, then the label data is placed back onto the pin and the specimen is refiled. A head pin is placed to mark where imaging should begin for future sessions.

The workflow below is described as if one person is digitizing (Fig. 3). If two people work together, one person can manage specimen related tasks and the second person can take photographs. The imaging process began with setting up the specimen. The specimen was taken out of its drawer, its labels removed from the pin, and placed on the gray foam board along with the new barcode label on the tempered glass. The specimen pin was inserted into a small piece of moldable silicone putty on the glass and the digitizer checked that the specimen’s wings were level. Labels were organized on the foam board in the following manner: scientific name was placed on top with locality labels directly below and miscellaneous labels either below or to the right of locality labels. Accession and catalog number labels were placed at the very bottom. While this is a general template on how labels are arranged, the organization differed when there were very large labels associated with the specimen. In such cases, labels were arranged so that all can fit within the area allocated for photography. The dorsal side was always imaged first because the “Datamatrix.py” script appended “_D” for the first image of a barcode. The camera was then adjusted so the specimen was in focus and a dorsal image was taken simultaneously in a .cr2 and .jpg. The specimen was turned over and the head of the pin was placed in the putty, so that the ventral side was facing the camera. Labels were checked to confirm that information was not written or printed on the back of the labels. If information on labels was present on the backside it was imaged in the ventral photo. The camera was focused again and an image was taken. In some rare cases, a third image was taken if there were large data labels. If the specimen was pinned laterally, only one image was taken. Image capture of laterally pinned was not standardized (e.g. consistently imaging either right or left side) and is an opportunity for further standardization. Data labels were placed back on the pin. The barcode label was pinned last and faced downward. The specimen was placed back into its original drawer or unit tray. At MGCL, we created a system so that at the end of the imaging session, the digitizer placed a colored pin in the insect drawer to denote where the image session was terminated. The scientific name, number of specimens imaged, and name of the digitizer were typed in a separate Excel spreadsheet to keep track of image metadata. Imaging equipment was cleaned and stored until next use.

### ﻿Pinned-specimen image processing

**Figure 4.**
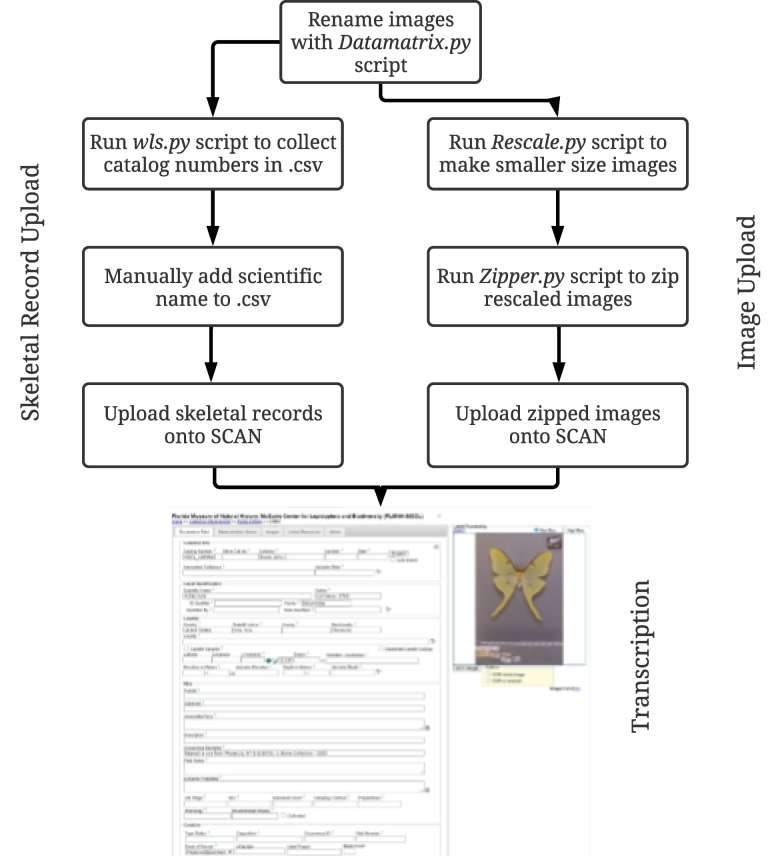
Post-processing workflow. Image processing begins with renaming barcodes using the *Datamatrix.py* script. The renamed images are then used to generate skeletal records and resize the images using the *Rescale.py* script and zipped using *Zipper.py* then uploaded onto SCAN. Skeletal records consist of the scientific name and the catalog number. Image resolution is reduced to thumbnail quality then zipped into one folder, before being uploaded onto SCAN.

Four Python scripts were utilized to process images (Fig. 4). The “Datamatrix.py” script processed an image to extract a specimen catalog number from a barcode. It renamed the image file to match the barcode data and used a directional indicator that rotated the image to a standardized vertical position. The script also appended the file name to note if the image was dorsal (“_D”), ventral (“_V”), or lateral (“_L”). An image of the dorsal side was taken before the ventral side, and the script used this consistency to rename images. Lateral images were renamed by hand because the default was for the first specimen of a unique barcode to be appended with “_D”. Less than one percent of specimens was renamed by hand because the barcode was unreadable. The “Rescale.py” script created a low resolution .jpg image from the original image, and the script, “Zipper.py”, zipped the downscaled images into a folder. The zipped images were uploaded onto SCAN. A fourth script, “wls.py”, generated a .csv file that listed all catalog numbers in each folder. The numbers were used to create a skeletal file which captured the scientific name and catalog number which was uploaded onto SCAN and linked to images. Scientific names were entered manually in the .csv file. Records with images and skeletal data on SCAN were then used for label transcription.

### ﻿Pinned-specimen transcription

**Figure 5.**
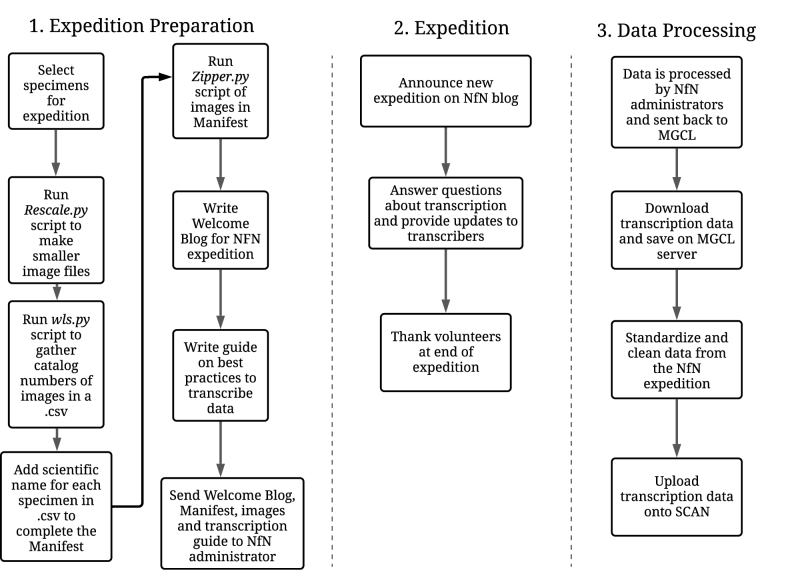
Notes from Nature workflow consists of three steps: preparing the expedition, maintenance during the expedition, and data processing. Preparation includes selecting specimens and images for transcription and writing a welcome blog and instructions on how to transcribe. During the expedition, we monitor for questions. Data processing consists of cleaning the data and formatting them so they can be uploaded into a database.

The main steps of an NfN expedition were specimen selection, creating a spreadsheet of data to be captured, and writing a paragraph containing information for volunteers about the expedition (Fig. 5). Expeditions had a particular theme, usually a taxonomic group to align with the needs of the institution or researcher. Once images were selected, they were downscaled using the “Rescale.py” script and zipped so that images were easier to upload to NfN. Before zipping, we made a Manifest: a .csv file that contained the catalog number and scientific name of specimens included in the NfN transcription expedition. To make the Manifest, we ran “wls.py” to capture the image file name in the folders. We manually added the scientific name and the catalog number to the .csv file, the latter of which was obtained easily from the image file name. If it was the first expedition in a series, we wrote a welcome blog, a paragraph explaining why these organisms are interesting, how data will be used and some eye-catching images. We included an instruction guide for transcribers with protocols of how to parse data and transcribe in a standardized method. The Manifest, images, welcome blog, and transcription instructions were sent to NfN via email. Once the expedition was launched, we announced the expedition on the NfN blog. We engaged transcribers throughout the expedition by answering questions and engaging in conversation. Transcribers were thanked at the conclusion of the expedition. Data from all three transcriptions per record were compiled by NfN staff, then sent to MGCL. Data in the reconciled .csv file were manually cleaned column by column to standardize data then uploaded onto SCAN.

### ﻿Statistics from LepNet digitization efforts

We tested our approach through the LepNet digitization efforts. Approximately 162,000 raw images of 81,000 specimens, representing ~2500 species of Lepidoptera, were digitized at the MGCL from September 2016 to May 2021. The number of images taken increased year over year from 9,872 in 2017, to 23,438 in 2018 to 37,209 in 2019. Most specimens were imaged twice (once dorsally and once ventrally). Although not a LepNet priority, on occasion, we imaged a laterally pinned specimen and its label data to enable data capture of the record. Specimens were imaged across 23 families with the majority in Erebidae (61,015 images), Saturniidae (37,301 images), Geometridae (18,903 images), and Sphingidae (17,761 images). Approximately 54,000 transcriptions (64% of LepNet specimens) were fully or partially completed. Of these 54,000 transcriptions, 21,500 were skeletal transcriptions (only genus, species, and country and/or state/province transcribed) and 32,500 were full transcriptions. The MGCL had 23 expeditions through Notes from Nature, totaling ~18,400 specimens. Of these 18,400 specimens, 10,400 were uploaded to SCAN. The remaining 8,000 needed data cleaning and were not included in the transcription total.

From February 2019 to May 2021 (27 months), MGCL personnel involved in the project collectively worked for 70,296 minutes (1,171.6 hrs) to obtain 54,099 images (includes dorsal and ventral), an average of 23 specimens per hour (sp/hr). The rate of imaging with one digitizer ranged from 11 to 23 per hour and was ~18 sp/hr while with two digitizers, the rate ranged from 13 to 44 per hour and was ~25–26 sp/hr. Forty-one community members and/or students volunteered. There were eight staff members, four of which were students who received hands-on training in a setting that usually has few opportunities for employment. Additionally, ~30 middle- and high-school students imaged and transcribed specimens for a week-long summer camp as part of the Florida Museum of Natural History’s Junior Volunteer program.

## ﻿Discussion

Digitization is often defined as a series of steps from pre-imaging to georeferencing (Nelson et al. 2012). Digitization requires decision making to optimize data-capture rate while maintaining desired data quality standards. Decisions related to the rationales and tradeoffs in a workflow are seldom discussed in papers. Our workflow was developed through extensive experimentation to determine the most effective equipment and settings for digitization and to evaluate their impact on reproducibility. This process involved iterative adjustments to equipment configurations and setup variables. Here, we discuss the benefits and drawbacks of the pinned-digitization workflow.

### ﻿Lightbox and copy stand

The combination of a lightbox and a copy stand for digitization creates a user-friendly and flexible method to image Lepidoptera specimens. The setup can accommodate most butterflies and large moths; increased magnification is required for imaging micromoths. Our digitization set up can image large specimens and specimens with unique morphologies such as those with long tails on their hindwings (e.g., some Saturniidae) by imaging on the glass surface or in the interior of the lightbox. This is due to the fact that the height of the camera mount on the copy stand can be adjusted. A lightbox and copy stand can be set up at minimal cost and can be used to generate images quickly. Once set up, the equipment is easy to maintain and use. The benefit of designing a customized lightbox to use in-house is that it can be made to desired optimal specifications, usually at a lower cost point. Lightboxes at MGCL were made of readily available supplies, including a wood frame coated in white paint, with LED strips glued and soldered on the inside for illuminating specimens during digitization. The main drawbacks with a copy stand in the MGCL digitization workflow centered around lighting. It is preferable to have all digitization stations in a single workspace, but in practice, it is often necessary to have digitization stations in different workspaces that inherently have variable lighting conditions. There are multiple sources of lighting to control, including copy stand lightbulbs, LED strips, and ceiling lighting. The MGCL had three digitization stations, each in a different room and with different ambient lighting, which required additional methods to ensure the accuracy of specimen coloration. For example, wax paper was put over the bulb fixtures in order to remove excessive glare of the light from the copy stand fixtures. Light- and color-calibration was performed with DataColor Spyder X Elite for each digitization computer monitor to standardize lighting between imaging stations. Another issue was that specimens of different sizes required different lighting angles and camera settings. Digitizers kept track of camera settings and the angles and heights of lights to standardize image capture throughout each project.

### ﻿Pinned-specimen imaging

The goal of the pinned-specimen imaging workflow was to capture specimen images and label data. For spread specimens, dorsal and ventral images were taken. For field-pinned specimens with unspread wings, a single lateral image was taken. We decided to image the dorsal and ventral sides of specimens because the ventral side of lepidopterans often has species-specific characteristics such as eye spots and other markings that are absent on the dorsal side. The main workflow decisions centered around specimen handling, data gathering, and speed and imaging efficiency.

We prioritized minimizing contact with specimens to ensure their long-term their preservation by handling specimens only once. To maximize the information obtained, we captured dorsal and ventral images along with associated label data. Although imaging both sides of specimens required more time initially, our approach reduced overall handling and proved more efficient in the long term.

We also had to decide whether to image label data separately or simultaneously with the specimen. Ultimately, we chose to capture label data simultaneously in images in both dorsal and ventral images. This approach was based on the idea that the images should aid in identification and analyses, rather than serve as publication-quality outputs, while also facilitating transcription. Another benefit of capturing the label data and specimen in the same image is that the photo and transcription can be checked against each other by future researchers. However, a drawback of this method is that the specimen occupies a smaller percentage of the image due to the inclusion of label data. One advantage of our setup is that information on data labels can be easily captured and does not require additional images to be taken. By standardizing the placement of label data, we aim to simplify the re-pinning process and reduce the need for searching for the desired label during transcription.

Data labels were placed on a platform ~0.75 inches tall, constructed from plank Styrofoam. This material was chosen for its durability – it resists breaking or denting while remaining malleable enough to accommodate pins. The elevated platform ensures that the label data and the barcode are in the same focal plane as the specimen.

Another consideration was whether imaging was more efficient solo or in tandem. A single digitizer averaged 18 sp/hr, which could be as high as 23 sp/hr if the digitizer was highly experienced. In contrast, two-person teams averaged 23–24 to upwards of 44 specimens per hour. The average difference in efficiency is minimal because, in tandem setups, one person may work faster than the other, leading to brief idle periods. However if two experienced imagers are working together the average could be significantly greater than normal. Tandem imaging offers benefits such as task specialization, allowing each person to focus on fewer responsibilities, and reducing feelings of isolation—an important factor for volunteers. Additionally, imaging efficiency varies with specimen size; smaller specimens are quicker to image than large specimens.

### ﻿Pinned-specimen digitization image processing

Images were processed every few weeks, with editing taking a few hours, primarily due to the time required for scripts to run. The “Datamatrix.py” script failed to rename some barcodes because there was debris on the barcode, a pinhole through the barcode, or part of the machine-readable barcode being either out of focus or missing from the image. In these instances, image files were renamed manually. For large Lepidoptera, such as *Attacus* L. spp. or *Antheraea polyphemus*, the camera had to be mounted higher, resulting in barcodes appearing too small for successful automated renaming.

### ﻿Pinned-specimen digitization transcription

Transcription in NfN had its advantages and disadvantages, with the primary considerations being time and quality. The NfN is a more time-intensive process for the expedition coordinator, requiring significant effort to prepare images for the expedition and to clean and upload the data afterward. Smaller projects may not justify the time investment, while larger projects risk prolonged completion times.

Transcriptions from NfN exhibited greater variability, requiring significant effort to standardize. This was because transcribers often entered label data exactly as it appeared, with fields like locality frequently abbreviated in inconsistent ways. To streamline formatting, we applied batch changes in Excel to standardize fields such as names, dates, and localities. For efficiency, we prioritized cleaning frequently repeated data, such as locality and collector names. Additionally, we added scientific names before the expedition to ensure accuracy and minimize transcription error. The focus on our project was developing the imaging infrastructure, and there are opportunities to improve the transcription workflow and better understand the efficacy of transcriptions from NfN. A future direction is to incorporate technologies and software to expedite cleaning. Options include OpenRefine software to clean and reconcile messy data (Delpeuch et al. 2024) or to incorporate Label Babel, recently developed machine learning optical character recognition methods integrated with NfN (Guralnick et al. 2024). Another emerging approach is to use artificial intelligence and large language models, to do the initial transcription then check these data by humans (Herbst et al. 2025).

### ﻿Fluid digitization on a copy stand

We conducted preliminary tests to assess whether a copy stand could be used to image an individual fluid specimen. In our tests, a specimen and data labels were removed from the jar and partially dried, then imaged on the copy stand (Suppl. material 1). We believe that this method may be useful to image individual specimens but requires further testing to optimize image quality and scalability of the method.

## ﻿Conclusion

We provide a workflow for digitization of Lepidoptera that covers pre-imaging preparation, imaging, image processing, and transcription. Our workflow utilizes a copy stand for pre-imaging digitization tasks and imaging pinned specimens and includes image processing and transcription steps, the latter two which utilize bioinformatic scripts for optimization. Our approach is fast and inexpensive compared to other existing digitization workflows, and it has been used in research projects, such as a study on *Catocala* Schrank wing color and polymorphism (Homziak 2022). Many entomology collections utilize copy stand workflows that have the same overarching steps but a myriad of ways to accomplish them. It is critical for global digitization success and efficiency to share open access workflows through publication, institutions, and community-driven resources. We hope that the sharing of workflows will further improve digitization best practices.

## ﻿Additional information

### Use of AI

No use of AI was reported.

### Author ORCIDs

Laurel Kaminsky https://orcid.org/0000-0002-0786-8350

Ngoc-Nhu V. Tran https://orcid.org/0000-0002-2555-2958

Anupama Priyadarshini https://orcid.org/0009-0003-2009-5940

David Plotkin https://orcid.org/0000-0002-2339-655X

Akito Y. Kawahara https://orcid.org/0000-0002-3724-4610

### Data availability

All of the data that support the findings of this study are available in the main text or Supplementary Information.

## Supplementary materials

Supplementary material 1 Notes on fluid digitization with a copy stand Data type docx Explanation note Observations of preliminary tests on how individual specimens removed from fluid can be digitized using a copy stand. Contains a workflow, at a conceptual level, for the process of specimen removal, imaging, and optimization of lighting and drying a specimen to minimize glare. https://binary.pensoft.net/file/1492453This dataset is made available under the Open Database License (http://opendatacommons.org/licenses/odbl/1.0/). The Open Database License (ODbL) is a license agreement intended to allow users to freely share, modify, and use this Dataset while maintaining this same freedom for others, provided that the original source and author(s) are credited.Laurel Kaminsky, Erin Jane Howard, Ngoc-Nhu V. Tran

Supplementary material 2 Supplies for pinned-specimen digitization Data type docx Explanation note List of supplies used in the copy stand digitization of pinned Lepidoptera for the McGuire Center for Lepidoptera and Biodiversity. https://binary.pensoft.net/file/1492454This dataset is made available under the Open Database License (http://opendatacommons.org/licenses/odbl/1.0/). The Open Database License (ODbL) is a license agreement intended to allow users to freely share, modify, and use this Dataset while maintaining this same freedom for others, provided that the original source and author(s) are credited.Laurel Kaminsky, Stacey Huber

Supplementary material 3 MGCL pinned-specimen digitization workflow Data type docx Explanation note Digitization workflow used to image pinned specimens. Contains in depth details on how to set up for imaging, image processing, data management, and upload onto SCAN. This document is aimed for the digitization manager and troubleshooting problems. https://binary.pensoft.net/file/1492455This dataset is made available under the Open Database License (http://opendatacommons.org/licenses/odbl/1.0/). The Open Database License (ODbL) is a license agreement intended to allow users to freely share, modify, and use this Dataset while maintaining this same freedom for others, provided that the original source and author(s) are credited.Laurel Kaminsky, Stacey Huber, Trudi Deuel, Ngoc-Nhu V. Tran, Erin Jane Howard, Aaron Leopold, Anupama Priyadarshini
