# Supplementary material for: ﻿A digitization workflow of dry-pinned collections of Lepidoptera
Source: Zookeys. 2025 Dec 15;1264:73–93. doi: 10.3897/zookeys.1264.134756 (PMC12723396; doi:10.3897/zookeys.1264.134756)
Supplement: Supplementary material 1 — Notes on fluid digitization with a copy stand [file zookeys-1264-073_article-134756__-s001.docx]

Supplement One: Notes on fluid digitization with a copy stand

Goal: To assess whether the copy stand workflow at MGCL could be used to image immature Lepidoptera specimens in fluid collections. We hope that these observations will be of interest to the digitization field, since we found no references of a similar technique.

Methods:

Fluid specimen digitization required two people with similar roles. One person handled specimen preparation by removing the specimen and label from the vial, partially drying the specimen, replacing fluid and/or the vial stopper if needed, and returning the specimen to storage. The second person was responsible for imaging. For fluid digitization, we conducted two imaging sessions with two digitizers, each with only two to three specimens to assess challenges for digitization. Trial 1 had two specimens, resulting in a total of four images being taken in about 10 minutes. In Trial 2, three specimens were imaged from three angles (lateral, ventral, dorsal) and their label data, resulting in a total of 12 images in about 32 minutes.

*Pre-imaging preparation of fluid specimens*

All specimens were stored in 70% ethanol in patent lip vials with rubber stoppers. We assessed whether the rubber stopper or fluid needed replacement based on signs of disintegration and discoloration of the rubber stopper, and pink or red discoloration in the fluid itself. Due to the exploratory scope of larval digitization, we imaged large specimens that had visible physical and color characteristics and legible label data. Larval digitization was conducted in a molecular lab, to allow for fluid replacement to be done in a fume hood. The copy stand was moved onto a bench adjacent to the fume hood. Ceiling lights in the room were turned off to set the white color balance.

*Fluid specimen imaging*

Fluid digitization begins with removing the rubber stopper that secures the opening of the vial. Label data and the specimen are removed with tweezers. The specimen is partially air-dried on a paper towel (for 30–60 seconds) before being imaged to reduce glare. The specimen is placed laterally on the light box with a ruler and color card, then imaged. When imaged with ceiling lights on, the glare obscured the details; turning the lights off greatly improved the image quality (Fig. SX.1). Labels are placed under two glass microscope slides and imaged separately. The fluid levels and the glass vial and rubber stopper quality are checked and replaced if needed. For consistency and ease in reading, the barcode is placed inside of the vial at the bottom with the information facing downwards. The specimen is then returned to the vial with the head facing the vial top. Lastly, labels are returned to the vial. The label is oriented so that its right side is inserted first, and the writing faces outward. After each specimen digitization, the digitizer types the scientific name and barcode in the Excel spreadsheet. The glass is cleaned, and the specimen is returned to the drawer.

*Discussion*

*Fluid specimen imaging on a copy stand*

We decided to put the specimen directly on a copy stand and lightbox to see how it would work for fluid digitization. Imaging proved tricky due to the slow imaging process and limitations of equipment. The two main issues with imaging were glare and ambient light. Specimens that were fully wet produced a glare and obscured color and morphology while those that were partially dried and placed directly on the glass did not. The ceiling lights were unevenly spaced and were a different wavelength and intensity than the bulbs in the copy stand, which created shadows and affected color temperature and the white balance. We decided to image specimens with all the ceiling lights off and to set the white balance on the camera by imaging a white piece of photocopy paper in the same lighting conditions as the specimens. This produced images with a gray background (Fig. 4). The specimen and labels were imaged separately because the labels were larger than the specimen.

Imaging larval digitization specimens with our setup would have some hurdles to overcome for optimization. The method requires at least two people because the pre-imaging and imaging tasks and re-curation are more time consuming than the respective pinned digitization tasks. Imaging took more time and caution because the specimens needed to be dried before imaging, which made them more fragile and required careful handling. Re-curation, such as replacement of fluid and/or vials was also necessary to preserve the specimen, but also added additional time. In addition, picture quality would need more testing. Large basic details were identifiable on the larvae, but smaller specimens would need a stronger lens or a different imaging setup. Also mostly dry, dark-bodied specimens often lacked details with white balance adjustment and will need further experimentation to bring out those details with imaging.

We decided that if larval digitization occurred in the future, a barcode would be placed on the outside of the vial, and in the fluid to serve as backup for the other in case of damage. Each barcode needs ink, and printing material (paper or plastic) best suited to their different position on or in the vial to ensure longevity. The challenge with using two barcodes is ensuring the same number inside and outside the vial is used.

*Imaging with 3D-printed boxes on a copy stand*

Before deciding to use the tempered glass on the copy stand for imaging of specimens in fluid we wanted to see if 3D printed boxes following Mendez et al. (2018) would work on the copy stand. Boxes measured 75 x 50 x 1 mm and were made from polylactic acid (PLA), printed on a Lulzbot T6 3D printer. We printed boxes in black, clear, white, and gray. Tests were conducted to see how box color affected the image quality. The first test with the copy stand was to image specimens inside a 3D-printed box on a copy stand using different color boxes. The color that worked best was gray, but the boxes were still too opaque. The box cast a shadow on the specimen, and the isopropanol increased glare. The shadows were cast because of the angled lights on the platform hitting the edges of the box, which created uneven shadows on the specimens. The larvae moved a lot in the fluid and required constant repositioning for imaging. With more fine tuning, we could probably get a 3D-printed box and copy stand workflow to work.


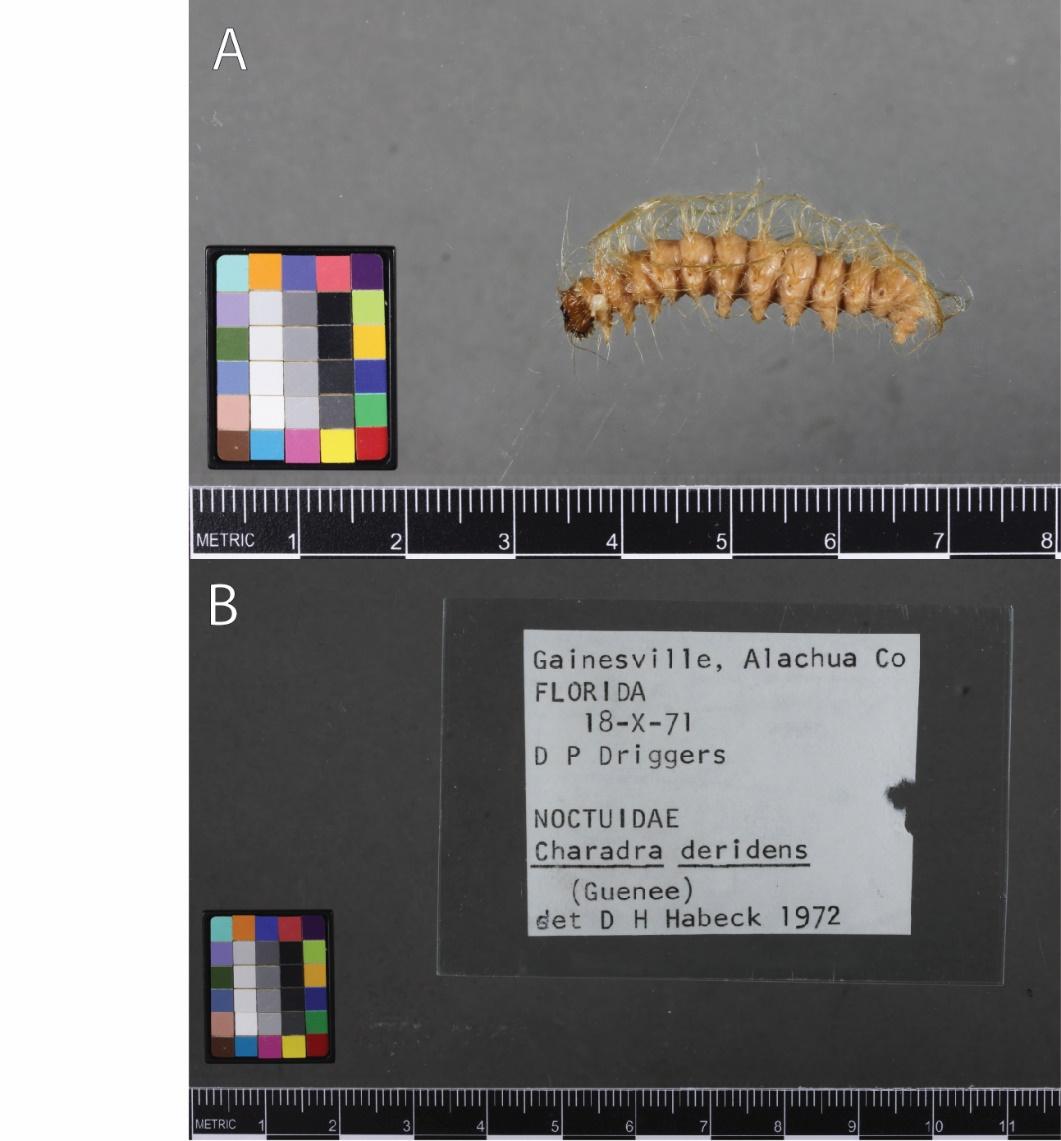


**Figure SX.1.** Example of larval images taken on the lightbox. **A** is a closeup of the specimen with the color card and **B** the associated label of the specimen.
