# Supplementary material for: ﻿A digitization workflow of dry-pinned collections of Lepidoptera
Source: Zookeys. 2025 Dec 15;1264:73–93. doi: 10.3897/zookeys.1264.134756 (PMC12723396; doi:10.3897/zookeys.1264.134756)
Supplement: Supplementary material 2 — Supplies for pinned-specimen digitization [file zookeys-1264-073_article-134756__-s002.docx]

**Supplies for Pinned Digitization at the McGuire Center for Lepidoptera and Biodiversity**

**Barcodes:**

BarTender- to make barcodes in house

Barcodes- code 128 on paper, printed with BarTender

Barcodes- data matrix on laminated barcodes, from AlphaSystems

**Camera supplies:**

Canon 18-55mm lens

Canon 60mm lens

Canon 7D body

Canon power adapter

**Copy stand:**

Copy stand- Beseler CS-14 copy stand

Lightbulbs- placed in copy stand fixtures

Wax paper- to dampen light from lightbulbs

**Lightbox:**

Glass plate/tabletop- to place specimens and label data on for imaging

LED lighting strips- placed inside the box for better lighting

Neutral gray plate- placed at bottom of lightbox for consistent background

White paint- to paint lightbox

Wood- to make lightbox

**Imaging:**

Color cards- to have record of color in photo

Silicone putty- to hold specimen pin

Foam board- plank foam board with neutral gray color, put label data on it
