# Supplementary material for: ﻿A digitization workflow of dry-pinned collections of Lepidoptera
Source: Zookeys. 2025 Dec 15;1264:73–93. doi: 10.3897/zookeys.1264.134756 (PMC12723396; doi:10.3897/zookeys.1264.134756)
Supplement: Supplementary material 3 — MGCL pinned-specimen digitization workflow [file zookeys-1264-073_article-134756__-s003.docx]

**MGCL Digitization Protocol**

**Camera Settings**

*Basement*: 1/8, F16, ISO 200

*Main Floor*: 1/8, F10, ISO 200

*Top Floor*: 1/8, F8, ISO 200

**Imaging Protocol**

1. Log into computer using UF credentials.
2. Once signed in, turn on the camera and all setup lights (two side lights plus the lightbox). Turning on the camera will automatically open EOS Utility on the computer. Once the program opens, select the *[****Camera Settings/Remote Shooting****]* option. Then select the *Live View Shoot* option. Position the color card so that it is in the top left corner of the image (note: the image gets rotated 90 degrees clockwise when it is saved, meaning that the color card should initially appear in the bottom left).
3. Navigate to the 1-IMAGES_ORIGINAL folder (/Lepidoptera/DIGITIZATION/PINNED_COLLECTION/1-IMAGES_ORIGINAL…)
4. From here, select the family that the specimens you are imaging belong to.
5. Create a folder containing the name of the genus of the specimen (if imaging in batches by genus, otherwise ignore this step).
6. Create your working folder. The name of this folder should be in the format: *Family_Genus_species(if applicable)_YYYY_MM_DD_AM/PM (time of day).*

Genus and species may be omitted in the folder name if imaging specimens from multiple genera. Copy this address by right-clicking the address bar, and paste it into the destination folder in EOS Utility (folder icon in top right of smaller window).

1. Remove specimens from collection drawer one at a time. Place in unit tray.
2. Pin a label containing the specimen genus and species in the upper left corner of the gray platform.
3. Using forceps, remove all pinned labels from the specimen. Place these labels onto the gray platform, either underneath or to the left of the genus/species information, as space permits. Add an MGCL data matrix label to the platform.
4. Pin the specimen, dorsal side up, into the putty on top of the glass so that the specimen is facing your left (will be facing upwards when the image is taken due to the rotation).
5. Slide the platform containing all labels so that it appears below the specimen in the final image.
6. In the larger camera window, click the *Live* button, which will focus the camera. You can change the focus location by dragging the box that appears over the image. After the focus box turns yellow, click the circular black button on the smaller window to take the picture (can alternatively just press the spacebar on the keyboard).
7. After confirming that the image has saved and everything is in the correct orientation, flip the specimen upside-down, so that the ventral side is now facing upwards. The head of the specimen should be facing the same way as before.
8. Take another image in this orientation.
9. Re-pin the labels to the specimen in the order and orientation that they were in previously. Try to pin the labels through the same pinhole that was already there; if this is too difficult or not possible, just be sure to avoid pinning through any data. Pin the MGCL data matrix last and facing away from the specimen, so that it can be read from underneath. Take care not to pin through the datamatrix.
10. Return specimen to drawer in same location it was retrieved from.
11. After imaging:
    1. Turn off camera and replace lens cover
    2. Place gray platform, forceps, and any other imaging material into unit tray
    3. Store the color card safely in its case
    4. Turn off all setup lights
    5. Update digitization logs:

/Lepidoptera/DIGITIZATION/1-IMAGES_ORIGINAL/DIGITIZATION_STATUS

**Image Processing Protocol**

1. Run the datamatrix-reader executable (Mac only). Instructions for dependencies and modularity can be found on the FLMNH-MGCL GitHub. Open the Images_CR2 folder where the executable is stored. Open a Terminal window (Command+Spacebar, type Terminal) and navigate to that folder using the following syntax:
   1. cd [*SPACE*] *N:\NaturalHistory\Lepidoptera\Kawahara\Digitization\LepNet\PINNED_COLLECTION\IMAGES_CR2*)
   2. *./datamatrix-reader [SPACE] --scan_time [SPACE] 30000 [SPACE] --start_dir [SPACE]* **[COPY AND PASTE FOLDER ADDRESS HERE]**

--scan_time refers to the amount of time (in ms) an image will be scanned. If no data matrix is found, the script will move to the next CR2/JPG pair after the allotted time. ****Note the double tag (-- vs -) for execution.**

1. Activate the Python virtual environment. This is currently located in Ryan’s staff folder under digitization/.venv[OS] (enable your file explorer to show hidden files by pressing Command+Shift+period on Mac, or going to View->Options->Change folder and search options->View->Advanced->Show Hidden Files on Windows). Activate the virtual environment via the following commands:
   1. Mac (Terminal)
      1. cd [*SPACE*] [**Copy and paste directory of .venvMAC file**]
      2. source [*SPACE]* tutorial-env/bin/activate
   2. Windows (PowerShell)
      1. cd [*SPACE*] [**Copy and paste directory of .venvWINDOWS file**]
      2. tutorial-env/Scripts/Activate.ps1
2. Run the wls script. All scripts from here on can be run on Windows (through PowerShell) or Mac (Terminal), but you will need the appropriate python libraries installed in the Python virtual environment. This script will parse all unique file names (MGCL numbers) into an excel sheet for processing. The syntax for this script is as follows:
   1. cd **[Copy and paste directory that contains the images]**
      1. **Windows:** python [*SPACE*] .\wls.py [*SPACE*] -u
      2. **Mac:** python3 [*SPACE*] .\wls.py [*SPACE*] -u

-u indicates that the script will only parse the unique MGCL numbers, irrespective of dorsal or ventral, CR2 or JPG

Open the excel file and clean the data manually (some rows in the first column will contain the directory the files originated from; these can be deleted). The only data that should remain in the first column is the unique MGCL number. Add a header for the column consistent with the database’s unique identifier (catalogNumber for SCAN). You can manually add column headers for other columns and add the associated data for each record (useful for genus/species information or quick pre-processing of country or collector data).

1. Run the Digitization console application. This is used to separate the JPGs from the CR2s, as well as downsize the JPGs. This can be found on GitHub (filename Digitization.py) or in Ryan’s staff folder *(/Digitization/LepNet/STAFF_FOLDERS*). The syntax to run is as follows:
   1. cd [*SPACE*] [**FILE DIRECTORY CONTAINING SCRIPT**], filename is digitization
   2. python (python3 if on Mac) [*SPACE*] .\Digitization.py (note the capitalization)
2. You will be greeted with the following menu:

Select program to load:

[1] Aiello Project

[2] Convert CR2 -> JPG

[3] Hlopp Project

[4] Legacy Upgrade Project

[5] Rename Project (Legacy)

[6] Rescale JPGs (Downscaler)

[7] Unique Value Tool (Database CSV)

[8] Zipper Tool

[9] Undo Tool

[10] MGCL Checker

[11] Batch Mover

or 'exit' to quit.

Select the appropriate program by entering the number in the braces. The relevant programs are [6] Rescale JPGs and [8] Zipper Tool

1. Rescale JPGs. Start by selecting if you’d like to see the usage information (generally no). Then copy and paste the directory that contains the images. This will be the folder that contains all the images whose filenames have been renamed to their respective MGCL numbers. Follow the instructions in the program. For upload to SCAN, we generally downscale the JPG size by a factor of 2, due to zipped file size limitations when uploading. Then select if you’d like to downscale only the images at that directory level, or all levels below if you have folders within a folder. After this option, the program will run and split the folder into 3 sub-folders—one containing all CR2s, one containing all hi-res (original) JPGs, and a third containing all of the downscaled images.
2. Zipper Tool. This program takes all of the downscaled images and zips them into a single zipped file with a set maximum capacity (default is 1 GB). Start by selecting if you’d like to see the usage information (generally no). Then it will ask for the directory that contains the files. Copy and paste the directory of the folder containing all of the images (CR2s, Hi-Res, and Low-Res). Next, the program will ask for the directory to save the zipped file to. This will always be */Digitization/LepNet/PINNED_COLLECTION/zipped_folder.* The next prompt will be about the capped size of the zipped file. If you’d like to keep it at 1GB, enter 1, otherwise enter 2 and follow the steps to manually set a cap.

**Uploading data and images to SCAN (https://scan-bugs.org/portal/)**

- - - 1. Log in to SCAN <https://scan-bugs.org/portal/>
      2. Click on 'My Profile' (top right), 'Specimen Management', 'Florida Museum of Natural History, McGuire Center for Lepidoptera and Biodiversity (FLMNH MGCL)'
      3. Under Administation Control Panel, click on 'Import/Update Specimen Records', 'Skeletal Text File Import' (if importing just a few key fields, such as catalog number, species name, country)
      4. 'Browse' to find CSV file, select it, then click 'Analyze File'
      5. Source fields should automatically map to target fields, otherwise manually select the appropriate target fields.
      6. Click on 'Upload'
      7. Click on 'Transfer Records to Central Occurrence Table'
      8. You will see the following, and an indication that the transfer is in progress:


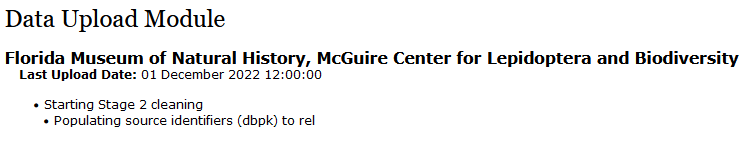


When the upload is complete (may take 5-10 mins) you will see:


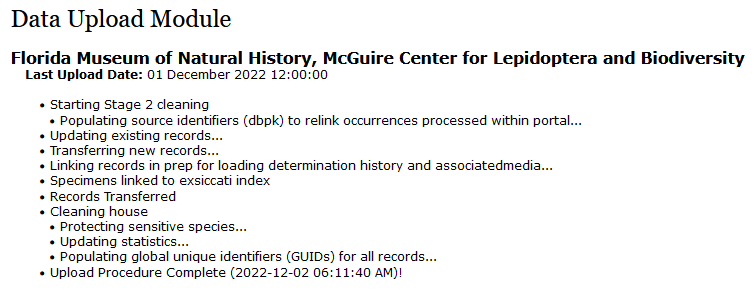


9. **Upload the images**. Sort images into folders, note that each image should be < 3 Mb, each zip file should be < 1 Gb, if more than 100 images in the zip file then upload over evenings or weekends. Click on 'Images', 'Batch Upload', 'Browse' for zip file and select it. Click 'Submit'.

10. You will get a log file on the webpage as follows, check that everything was completed successfully for each image. NOTE: records and images may not be visible when you Search in SCAN for several hours after uploading.

[2022-12-02 06:27:48am][INFO] Upload for MGCL_upload_221202_1.zip succeeded

[2022-12-02 06:27:48am][INFO] Starting image processing on MGCL_upload_221202_1.zip...

[2022-12-02 06:27:48am][INFO] Found MGCL_upload_221202_1

[2022-12-02 06:27:48am][INFO] Catalog number not found in MGCL_upload_221202_1/. Skipping...

[2022-12-02 06:27:48am][INFO] Found MGCL_1104247_D.JPG

[2022-12-02 06:27:48am][INFO] Catalog number is MGCL_1104247

[2022-12-02 06:27:48am][INFO] Extracting MGCL_1104247_D.JPG...

[2022-12-02 06:27:48am][INFO] Found associated occurrence: Heraclides cresphontes

[2022-12-02 06:27:48am][INFO] Linking image to occurrence...

[2022-12-02 06:27:48am][INFO] Image imported successfully

[2022-12-02 06:27:49am][INFO] Thumbnail created successfully

[2022-12-02 06:27:49am][INFO] Large image created successfully

[2022-12-02 06:27:49am][INFO] Successfully uploaded image for occurrence 64295697: scan-bugs.org/imglib/scan/misc/202212/MGCL_1104247_D.JPG

11. Repeat to upload all the zipped image folders.
